# Again and Again—Survival of *Candida albicans* in Urine Containing Antifungals

**DOI:** 10.3390/pharmaceutics16050605

**Published:** 2024-04-29

**Authors:** Nevio Facchini, Lukas Wernli, Malte Rieken, Gernot Bonkat, Dieter Wirz, Olivier Braissant

**Affiliations:** 1 Department of Biomedical Engineering, University of Basel, Hegenheimermattweg 167B/C, 4123 Allschwil, Switzerland; nevio.facchini@stud.unibas.ch (N.F.);; 2 Faculty of Medicine, University of Basel, Klingelbergstrasse 61, 4056 Basel, Switzerland; 3 Department of Urology, Kantonsspital Baselland, Rheinstrasse 26, 4410 Liestal, Switzerland; 4 alta uro AG, Centralbahnplatz 6, 4051 Basel, Switzerland

**Keywords:** *Candida albicans*, urinary tract infection, isothermal calorimetry, relapse and persistence, antifungal agents, fluconazole, flucytosine, autophagy, blastospores, chlamydospores

## Abstract

Background: Relapse of *Candida albicans* urinary tract infection (UTI) is frequent despite appropriate treatment, as commonly used antifungals such fluconazole and flucytosine are only fungistatics. To improve treatment of *Candida* UTI and decrease relapses, understanding the long-term metabolic activity and survival of *C. albicans* in urine containing antifungals at minimal inhibitory concentration (MIC) is needed. Methods: we monitored the survival, metabolic activity and consumption of glucose and proteins by *C. albicans* using conventional methods and isothermal microcalorimetry (IMC). We also investigated the influence of dead *Candida* cells on the growth of their living counterparts. Results: For 33 days, weak activity was observed in samples containing antifungals in which *C. albicans* growth rate was reduced by 48%, 60% and 88%, and the lag increased to 172 h, 168 h and 6 h for amphotericin, flucytosine and fluconazole, respectively. The metabolic activity peaks corresponded to the plate counts but were delayed compared to the exhaustion of resources. The presence of dead cells promoted growth in artificial urine, increasing growth rate and reducing lag in similar proportions. Conclusions: Even with antifungal treatment, *C. albicans* relapses are possible. The low metabolic activity of surviving cells leading to regrowth and chlamydospore formation possibly supported by autophagy are likely important factors in relapses.

## 1. Introduction

Candiduria is a common finding. Up to 20% of hospitalized patients may have candiduria throughout their hospitalization, particularly patients in intensive care units [1]. In most cases, it merely represents colonization or contamination instead of urinary tract infection (UTI) [2]. In contrast to candiduria, which is frequently asymptomatic, *Candida* UTIs can have severe consequences including pyelonephritis, chronic renal failure, candidemia and even death [2]. The Infectious Diseases Society of America (IDSA) has published guidelines for the treatment of *Candida* UTIs [3]. The strength of these recommendations is based primarily on clinical experience, opinions of authorities, descriptive studies and a single randomized, double-blinded placebo-controlled trial [4]. In the latter, candiduria was either treated with 200 mg fluconazole daily or placebo for 14 days. An overall statistically significant response rate of 50% with the drug versus 29% with placebo was noted. However, 2 weeks after treatment had ended, both placebo-treated and fluconazole-treated patients manifested similar rates of candiduria, emphasizing the propensity of *Candida* spp. for both persistence and relapse in the urinary tract [4]. Candiduria might be related to (i) predisposing factors (e.g., indwelling urinary catheters, urine stasis, diabetes mellitus, extremes of age, immunosuppressive therapy, etc.), (ii) known and unknown intrinsic candidal resistance mechanisms, and (iii) pharmacokinetic and pharmacodynamic characteristics of the antimicrobials used [2]. In fact, except for amphotericin B, there is no commonly used true fungicidal drug available. Although some echinocandins have shown fungicidal activity, they are not excreted in urine and thus are of limited use [5]. The widely used azole derivates such as fluconazole or pyrimidine analogues like flucytosine are clearly fungistatic [6,7]. In the setting of *Candida* UTIs, processes such as umbrella cell exfoliation, neutrophil influx and urine flow will then contribute to “flush” such inhibited yeast cells. However, with respect to the risk factors described above, one needs to keep in mind that urine stasis, often due to long-standing diabetes or other neurological problems resulting in a hypotonic bladder, might severely impair such processes [8,9]. Considering that between 7.0 and 11.2% of adults (between 18–99) are suffering from diabetes [10], keeping such information in mind, one can hypothesize that patients with diabetes, the immunocompromised and/or patients with neurological disorders might be at higher risk of persistence and/or relapse of *Candida*. This emphasizes that both the choice of an antifungal agent and therapy duration might be crucial.

As currently no data on the survival of *Candida albicans* exposed to antimicrobials in urine are available, observation of the long-term metabolic activity, survival and relapse or regrowth of *Candida* spp. is of interest. Such data might not only be used to optimize the duration of antifungal treatment in *Candida* UTIs and candiduria but also to gain new insights into the physiology of *Candida* spp. In this study, we monitored the long-term metabolic activity and survival of *C. albicans* in artificial urine containing different commonly used antifungal compounds (i.e., fluconazole, flucytosine and amphotericin B) by using the sensitivity of isothermal microcalorimetry (IMC) [11,12,13,14]. In addition, as autophagy has been pointed out as a potentially important factor that could play a major role in the survival of *C. albicans*, the influence of autophagy on the relapse and persistence of candida infections was further investigated. Indeed, autophagy might explain the persistence of *C. albicans* after the exhaustion of resources and allow longer survival when facing antifungals by providing the necessary resources to build blastospores and/or chlamydospores and to run efflux pumps. As this aspect has not been investigated yet, we investigated the influence of the presence of killed *Candida* cells on the growth of *Candida* in artificial urine as well.

Overall, this study hypothesizes that *C. albicans* can survive at minimal inhibitory concentrations (MICs) of fluconazole, flucytosine and amphotericin B and maintain active metabolism over a rather long period (30 days in our study). Those antifungals were chosen because of their common use and their different mechanisms of action: Fluconazole inhibits the formation of ergosterol. Flucytosine is converted into 5-fluorouracil within yeast cells that is incorporated into RNA, causing the inhibition of protein synthesis. Finally, amphotericin B has a high affinity for ergosterol and binds to it in the fungal cell wall, causing membrane permeability and leakage of the cytoplasmic contents [15]. Furthermore, we also hypothesize that *C. albicans* can benefit from the presence of dead sibling cells to grow and promote relapse (thus mimicking autophagy). We use IMC and conventional biochemical assays to test those hypotheses, as IMC is well suited for measuring low metabolic activities using metabolic heat.

## 2. Materials and Methods

### 2.1. Candidal Growth and Microcalorimetric Measurement

Isothermal microcalorimetry was used to measure the heat produced by the metabolic activity and growth of *C. albicans* (DSM1665—obtained from the Deutsche Sammlung von Mikroorganismen und Zellkulturen (DSMZ)) in artificial urine with and without antifungal agents. All chemicals needed for the preparation of artificial urine were obtained from Sigma-Aldrich Co. (St. Louis, MO, USA) and the artificial urine was prepared according to the formulation of Brooks and Keevil [16], that is: peptone 1.0 g·L^−1^, yeast extract 5.0 mg·L^−1^, lactic acid 0.1 g·L^−1^, citric acid 0.4 g·L^−1^, NaHCO_3_ 2.1 g·L^−1^, urea 10.0 g·L^−1^, uric acid 0.1 g·L^−1^, creatinine 70.0 mg·L^−1^, CaCl_2_·2H_2_O 0.37 g·L^−1^, NaCl 5.2 g·L^−1^, FeSO_4_·7H_2_O 10.0 mg·L^−1^, MgSO_4_·7H_2_O 0.5 g·L^−1^, NaSO_4_·10H_2_O 3.2 g·L^−1^, K_2_HPO_4_ 1.2 g·L^−1^, KH_2_PO_4_ 1.0 g·L^−1^, NH_4_C1 1.3 g·L^−1^. To enhance pathogen growth and thus detection, 20 mg·L^−1^ of lactose, 20 mg·L^−1^ of saccharose and 560 mg·L^−1^ glucose were added to the artificial urine. The pH of the artificial urine was adjusted to 5.7 using HCl 2 M. The artificial urine was filter-sterilized using a 0.2 µm pore size stericup (Millipore, Darmstadt, Germany) and stored in the fridge for no more than 1 week. The glucose concentration used in the artificial urine was dictated by previous work emphasizing that such a concentration was found to provide a fair trade-off between a reasonable glucose concentration (normal (i.e., nondiabetic) human urine (30–200 mg L^−1^)) and a strong calorimetric signal allowing precise MIC determination. Furthermore, such a concentration remains well below commonly accepted glucose concentrations used in artificial urine ranging from 3 to 80 g·L^−1^ (see [17,18,19]).

All drugs were purchased from Sigma-Aldrich Co. (St. Louis, MO, USA). The minimal inhibitory concentrations (MICs) of antifungals used were determined in previous studies, and were as follows: Amphotericin B 0.5 µg·mL^−1^, Fluconazole 0.8 µg·mL^−1^, Flucytosine 5 µg·mL^−1^ [2,12,20,21,22].

For microcalorimetry, plate count protein determination and glucose measurements, sterile 4 mL microcalorimetric ampoules were filled with 3 mL of artificial urine containing the above-mentioned antifungal concentrations. Ampoules without antifungal agents were prepared as the control group. Then, all vials were inoculated with 20 µL of yeast suspension. The inoculum was prepared by diluting previously prepared and frozen (at −80 °C) 100 µL aliquots containing ~7.9·10^7^ CFU·mL^−1^ in 3 mL of artificial urine. Plate counts performed on YPD medium (yeast extract 10 g·L^−1^, Peptone 20 g·L^−1^, Dextrose 20 g·L^−1^, agar 15 g·L^−1^, pH 6.5) indicated that each vial was inoculated with ca 5.5·10^4^ ± 4.5·10^2^ colony-forming units (CFUs). All chemicals needed for the preparation of YPD medium were obtained from Sigma-Aldrich Co. (St. Louis, MO, USA).

After inoculation, the ampoules were crimp-sealed using a metal cap with a silicone rubber seal, the remaining headspace being ambient air. Following sealing, four ampoules of each group were introduced to a TAM48 (Waters/TA) microcalorimeter equipped with 48 channels, previously set and equilibrated at least for two days at 37 °C. For each channel, the heat production rate (heat flow expressed in µw = µJ·s^−1^) was measured in real time.

The remaining vials were incubated in an oven at 37 °C for the duration of the experiment. Vials were sacrificed (opened and not reused) at regular intervals to perform CFU counts, glucose measurements and protein determination. Prior to any measurements, the vials were shaken for at least one minute to homogenise the sample. All measurements were performed using 3 different vials (i.e., 3 replicates). Previous experiments using IMC have shown that candidal growth occurred in the first 24 h. Therefore, to obtain an accurate growth curve in the control group (i.e., without antifungal agents), we sampled vials to perform a plate count every three hours for the first 24 h. Then, all groups were sampled every 24 h for the first three days, and a daily plate count was performed for every group. Afterwards, every three days, a plate count was conducted. All plate counts were performed in triplicate. Serial dilutions were performed in PBS to obtain plates bearing between 20 and 100 CFUs. Along with every plate count, one millilitre of the sample was stored at −80 °C for the determination of protein and glucose concentration at the end of the experiment. Negative controls were performed by using uninoculated samples.

### 2.2. Determination of Protein Concentration and Glucose

Dissolved protein concentration remaining in the medium was determined using the bicinchoninic acid assay [23] (BCA assay—Carl Roth—Arlesheim, Switzerland) after sample filtration through a 0.2 µm syringe filter. For this assay, a standard curve was constructed using bovine serum albumin (BSA). Glucose was determined enzymatically using a glucose meter (Abbott, Precision Xceed, Chicago, IL, USA). To verify the accuracy of the glucose meter, a standard curve was constructed adding known amounts of glucose to artificial urine. Similarly, for the determination of low concentrations of glucose, samples were doped with known amounts of glucose [24,25].

### 2.3. Autophagy

Using the same methods, experiments were conducted to observe the potential role of autophagy in the persistence of *C. albicans*. Dead *Candida* cells were produced by autoclaving a concentrated culture grown in YPD (yeast extract 10 g·L^−1^, Peptone 20 g·L^−1^, Dextrose 20 g·L^−1^, agar 15 g·L^−1^, pH 6.5), and rinsed twice with PBS (Phosphate-Buffered Saline) to wash away the nutrient and collect the yeast cells. After autoclaving (105 °C), the killed *Candida* cells (0%, 0.33%, 1%, 5%, final concentration of the autoclaved cultures) were added to artificial urine inoculated with living *Candida* cells (10^4^ CFU·mL^−1^). All measurements were performed in triplicate and the experiment repeated twice.

### 2.4. Microscopy

After 7 days (for controls) and 31 days (for antifungal-containing samples), samples were observed using an Olympus microscope (Provis AX70, Olympus AG, Volketswil, Switzerland). For the observation, 10 µL of culture was deposited on a glass slide and covered with a coverslip.

### 2.5. Statistical Analyses

Using the microcalorimetry data, the growth rate (µ IMC) and the lag phase duration (λ) were calculated by fitting the Gompertz model over the heat data using R statistical software 3.0.1 and the grofit package [26,27]. Due to the lower amount of data, the growth rate (µ CFU) was calculated by fitting an exponential model (N_t_ = N_0_·e^µt^) over the plate count data.

## 3. Results

**Figure 1:**
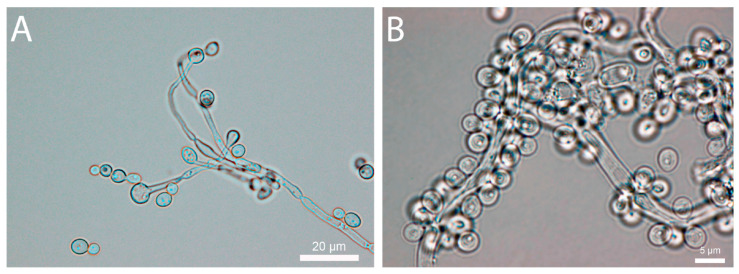
Microscopic observation of *C. albicans* grown in artificial urine with and without antifungal compounds. (**A**) Pseudomycelium of *C. albicans* with terminal chlamydospores. (**B**) Large number of blastospores associated with a pseudohyphea of *C. albicans*.

After inoculation, colony counts of different experimental conditions were between 1.4·10^4^ and 2.2·10^4^ CFU·mL^−1^, thus matching closely the inoculum size determined (see above). Monitoring took place over 33 days. During this time, the metabolic activity determined using IMC generally showed a main peak that appeared either slightly before or at the same time as the peak in CFUs. Similarly, the metabolic heat production peaks were observed when the highest consumptions of glucose and protein were observed. Interestingly, in all treated samples, metabolic heat production was maintained long after glucose and protein present in the artificial urine had been exhausted. At this point, it must be noted that metabolic by-products have not been investigated. Metabolic heat production and CFU counts were used to determine the growth parameters of *C. albicans* under the different conditions tested (Table 1). As expected, the growth rate of the control was higher than the growth rate in samples containing antifungals. However, when calculating growth parameters using plate count data and microcalorimetric data, we observed that the plate count data led to a higher growth rate (Table 1). Formation of blastospores and chlamydospores is believed to be the main driver for such discrepancies. Under low-glucose conditions, the formation of metabolically inactive spores is a well known process in *Candida* cultures [28]. As microcalorimetry only detects metabolically active cells generating heat, it will not detect those spores. On the other hand, once plated on rich medium (YPD), the *Candida* chlamydospores will be able to germinate again and produce a colony, thus leading to a higher growth rate. Indeed, microscopic observations confirmed the presence of large amounts of pseudomycelium and associated blastopores and chlamydospores (Figure 1).

### 3.1. Controls

**Figure 2:**
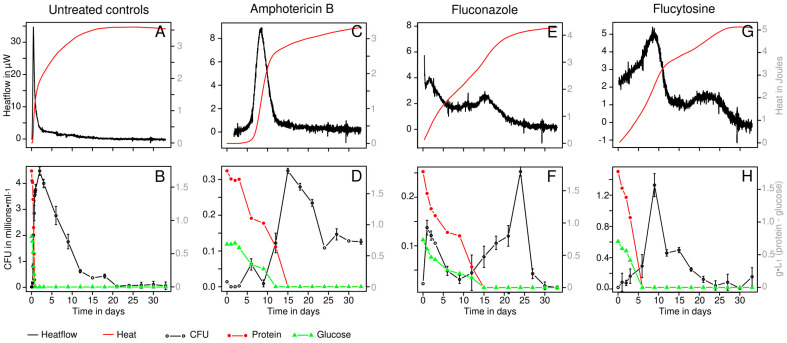
Microcalorimetric (heat flow and cumulative heat) and biochemical measurements (CFUs, protein concentration and glucose concentration) during the growth of *C. albicans* in artificial urine without antifungal compounds (**A**,**B**) as well as with Amphotericin B (**C**,**D**), Fluconazole (**E**,**F**) and Flucytosine (**G**,**H**) at predetermined MIC levels. Isothermal microcalorimetry show a representative curve. Biochemical and CFU measurements are the average of 3 replicates. Coefficients of variation for glucose and protein were 4% and 3%, respectively. Error bars for CFUs represent the standard deviation (the absence of error bars indicate an error bar smaller than the symbol).

In the controls, a steep increase in heat flow for the first 12 h with a peak reaching 36 µW was detected. Then, a gradual decrease in heat flow for the next 36 h was observed (Figure 2). Similarly, protein (i.e., 1742 µg·mL^−1^) and glucose (i.e., 757 mg·L^−1^) present in the medium were fully consumed during the first 21 h. The concentration of *C. albicans* rose up from 2·10^4^ to 4.5·10^6^ CFU·mL^−1^ within 48 h. The timeframe with elevated heat flow perfectly covered the time of growth defined by colony count. During the next ten days a continuous decrease was observed until the cell concentration reached 6.2·10^5^ CFU·mL^−1^. After this time point, the concentration of cell decreased more slowly (Figure 1). After day 21 *C. albicans* concentration was comprised between 4.6·10^4^ CFU·mL^−1^ and 1.1·10^5^ CFU·mL^−1^. The cell concentration measured during this final part was roughly comparable to those observed in samples containing antifungals (Figure 2). The growth rate measured by plate count was higher (0.147 h^−1^) compared to the growth rate estimated using the calorimetric data (0.078 h^−1^) as for all samples.

### 3.2. Antifungal Treatments

Samples treated with antifungals showed weak but still detectable candidal growth. Overall, far fewer CFUs were counted in these samples. IMC revealed a much lower metabolic activity (maximum peaks between 4 and 9 µW) and also a slower growth rate (between 0.014 h^−1^ and 0.037 h^−1^—Table 1). Again, the growth rates determined by CFU counts were higher than those calculated using calorimetric data (Table 1). However, the metabolic activity peaks observed by microcalorimetry coincided rather well with the peak in CFUs measured by the plate counts. In general, the consumption of protein and glucose occurred at a slower rate, but still showed a good correlation with the conventional and the microcalorimetric data. However, in all cases, the whole amount of glucose and protein were consumed, resulting in roughly similar amounts of heat produced (4.5 ± 1.0 J—Figure 2A–H).

Amphotericin B-treated samples showed almost no metabolic activity for the first 4 days. Similarly, neither glucose nor proteins were consumed from the medium. This is in line with the CFU counts, showing that out of the 1.4·10^4^ CFU·mL^−1^, no measurable survival of *C. albicans* was observed (i.e., CFU counts were below the detection limit of 10 CFU·mL^−1^, showing that more than 99.9% of the cells were inactivated/killed). Thereafter, a metabolic activity peak was clearly visible from day 5 to day 13 (Figure 2C,D), again matching well with the consumption of protein and glucose. In parallel, continuous candidal growth was observed until day 15. At this point, approximately 3·10^5^ CFU·mL^−1^ were found in the medium.

Flucytosine showed the largest candidal growth despite its presence at MIC concentrations. Metabolic activity increased until day 9 up to 5 µW; however, at day 6 the glucose and the protein were already depleted from the medium. In contrary to amphotericin B, no initial reduction in CFU·mL^−1^ was detected, and the yeast concentration after 9 days was ca 1.3·10^6^ CFU·mL^−1^ (Figure 2G,H). This was the highest amount of cells produced in antifungal-containing samples.

Fluconazole also showed no initial reduction in *C. albicans*. However, it featured the lowest peak in metabolic activity (ca. 4 µW) and maximal cell concentration, with only 2.5·10^5^ CFU·mL^−1^ after 24 days and low colony counts throughout the whole experiment (Figure 2E,F). Protein and glucose consumption were also the slowest, with these resources being exhausted at day 15 only. Noteworthy, with fluconazole, two activity and cell count peaks were observed. Similarly, the consumption of glucose and protein from the artificial urine clearly proceeded in two steps.

### 3.3. Proxy for Autophagy (Growth with Added Dead Cells)

**Figure 3:**
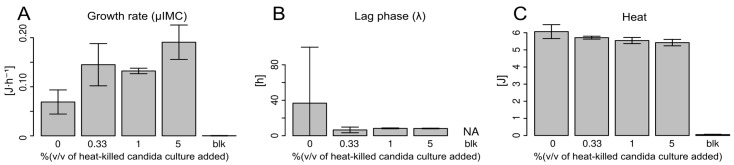
Bar plots of the samples containing living *Candida* cells (10,000/mL) and a variable concentration of autoclaved *C. albicans* cells (0%, 0.33%, 1%, 5%) showing the effect of the dead cell on the growth rate (**A**), lag phase duration (**B**) and metabolic heat production (**C**) of the living ones. For the statistical analyses, the results were computed using the Gompertz model and the variables analysed were growth rate (growth rate), lag phase (beginning of exponential growth) and Q (heat produced). NA: not applicable, blk: blanks (i.e., uninoculated controls).

The samples with a higher concentration of dead *Candida* exhibited a higher growth rate and a much shorter lag phase (Figure 3A,B). A minimal concentration of autoclaved *Candida* cells (i.e., 0.33%) was sufficient to achieve a significantly lower lag phase. At all concentrations tested, the lag phase value remained rather constant, emphasizing that the presence of dead *Candida* cells, even in very small amounts, enhances the growth of their living counterparts. The heat produced remained stable during the course of the experiment (Figure 3C).

## 4. Discussion

Combining conventional methods with isothermal microcalorimetry allows us to follow the concentration and the metabolic activity of *C. albicans* in urine as well as the concentration of available resources. In this context, isothermal microcalorimetry offers real-time monitoring of very low metabolic activity with a high time resolution that would otherwise require work-intensive assays such as tetrazolium-based assays or ATP assays that might even not be sensitive enough for such concentrations of yeasts [29,30]. Interestingly, in controls and samples treated with fluconazole or flucytosine, when resources (glucose and proteins) are exhausted, metabolic activity can be maintained up to 20 days even in the presence of antifungals at MIC levels. In these examples, the survival of *C. albicans* is not linked to a quiescent or dormant state but rather to a metabolically active state. Because the medium is rather poor (i.e., not many nutrients), it is rather unlikely that such activity would be sustained by reserve molecules (such as polyphosphate) or by by-products. Still, this should be further investigated to completely rule out such mechanism. Similarly, as in most cases, the cell number still increases when nutrients are depleted, and thus it is also unlikely that additional nutrients might come from lysed *Candida* cells. On the other hand, autophagy might allow the reallocation of the necessary resources to withstand the starvation period [31,32,33]. In addition, we can hypothesize that autophagy might also provide some energy to drive the efflux of fluconazole and flucytosine, thus allowing not only survival of starvation but of antifungals as well. All these observations are consistent with the fungistatic action of both fluconazole and flucytosine but also need to be considered in the light of the higher concentration excreted in urine during patient treatment (>100 μg·mL^−1^ for fluconazole and >30 μg·mL^−1^ for flucytosine) that are clearly above the MIC observed for susceptible *Candida* species [34]. We are well aware that using dead cells as a proxy for autophagy has strong limitations, mostly because the link between autophagy and dead biomass is very indirect as living cells could also contribute to autophagy. Indeed, further studies could provide more detailed results by using incorporation assays with either stable isotopes or analogue substrates combined with click chemistry, for example [25].

Amphotericin B was the only antifungal for which the metabolic activity stopped after resources were fully consumed. As expected, amphotericin B was also the only agent with a fungicidal effect (i.e., showing an immediate net decrease in the CFU number). For two days, no colonies could be detected. The later growth of *C. albicans* proves that some yeasts survived, showing that a 100% reduction in pathogens failed at MIC. At this point, one must remember that no growth after 48 h would be considered as true inhibition in most clinical settings. The regrowth starting at day 6 could be explained by the presence of fungal microclusters allowing only poor access of the drug to their centre [35]. As for fluconazole and flucytosine, concentrations of amphotericin higher than MIC are expected in urine (up to 4 μg·mL^−1^). Also, the excretion has been shown to last over days [36,37,38]. These pharmacokinetic data should also be taken into account for further investigations.

With respect to clinical practice, it is useful to divide patients with *Candida* UTIs into complicated and uncomplicated cases. Immune-competent patients without chronic diseases can be treated with oral fluconazole as current guidelines recommend [4]. Amphotericin B bladder irrigation is also used in some cases; however, this treatment is subject to debate [4,39]. Intravenous use of Amphotericin B tends to cause toxicity. Therefore, it is predestined to use it in more complicated cases. Such complicated cases are marked by additional factors like immune suppression, in-patient stay, malfunctions of the urine flow, etc. In these cases, relapses are much more frequent and the use of amphotericin B, prolonged fluconazole treatment or a combination of both is advised. Indeed, our results suggest that early use of amphotericin B to reduce the number of pathogens [39,40], followed by fluconazole to avoid the regrowth of the survivors, could be a good approach [4,39,40]. As recommended in the literature, the use of flucytosine alone should be avoided, especially considering the advantages of combining flucytosine and amphotericin B for the treatment of complicated cases [41]. Antifungal therapy mostly relies on a limited number of compounds. Among those, azole derivatives represent a rather large proportion; however, azoles, as demonstrated, are mostly fungistatic. Similarly, other antifungal compounds such as flucytosine and griseofulvin are also fungistatic. For *Candida* spp. and potentially many other fungal pathogens, it would make sense to investigate the combinations of such antifungals with fungicidal compounds such as allylamine (terbinafine, for example), polyene (amphotericin B), echinocandin (Anidulafungin, caspofungin, and micafungin) or bore-containing compounds (oxaboroles, for example) [41,42]. As echinocandins are not excreted in urine [6], application could be performed through bladder irrigation as a replacement for the more toxic amphothericin B.

Several risk factors for *Candida* cystitis have been identified which also increase the risk of relapse. Among these risk factors are, among others, increasing age, female sex, diabetes mellitus, prolonged hospitalization, intensive care unit stay, immunosuppressive therapy, recent use of broad-spectrum antibiotics, previous urological surgery, radiation therapy, abnormalities of the urinary tract and catheterization [2,43]. Given these factors, various reasons for relapses may be considered, including host immunity, biofilm formation or a dysfunctional glycosaminoglycan layer of the bladder epithelium. Although urinary tract obstruction as well as biofilms on catheters play an important role as risk factors for infection and relapse, other factors have to be considered as well. In this context, any treatment of the predisposing factors will improve the chances of a successful treatment of a *Candida* urinary tract infection. Nevertheless, as we see relapses after successful eradication of *Candida* in the urine, the mechanisms observed in our study may provide an additional explanation for the recurrence of *Candida* urinary tract infections and warrant further investigation.

Additionally, the autophagy of *C. albicans* needs to be further studied. A substantial yet unexpected difference in composition of the autophagy machinery between *C. albicans* and other species has been already discussed in other studies [44]. Our results show that autophagy may play an important role in *Candida* relapses, probably even after effective antifungal therapy [44]. Indeed, very few dead cells were sufficient to trigger a strong increase in growth rate and decrease in the lag phase. A deeper understanding of the phenomenon could allow research into new strategies to control relapses in high-risk patients such as the immunocompromised. Autophagy could also be related to chlamydospore and blastospore formation. The mechanism which allows *Candida* to sporulate is not fully understood yet, but still many factors have been discovered that can influence the phenomenon [45,46]. In particular, azoles seem to enhance the production of farnesol [47], which appears to be linked to the production of chlamydospores [48].

Another important topic to explore in the coming years could be biofilm formation. This mechanism might also participate in the complex functioning of *C. albicans* relapse and persistence. In this context, our system shows limitations, mainly due to the static and closed nature of our setup. The use of closed microcalorimeter vials results in the consumption of oxygen and promotes shifts from aerobic to anaerobic metabolism. Although urine is usually not saturated in O_2_ with respect to atmosphere, this could influence our results [49,50]. Furthermore, closed vials also do not allow urine flow to be mimicked. Such limitations could be overcome using flow-through systems (conventional flow cells or flow-through calorimeters); these would allow a fresh medium input and *Candida* flushing closer to urinary tract conditions, but at the cost of a lower throughput [51].

Finally, in addition to the technical limitations imposed by the calorimetric studies discussed above, we also have to recognize other limitations. In particular, the use of heat-killed cells is suboptimal as the heat denaturation leads to cell debris that is probably different from cells damaged by antifungals. Future studies should use more appropriate procedures, such as peracetic acid inactivation, which is commonly used to produce vaccines [52]. In addition, despite our promising findings, we have to acknowledge that only one strain was investigated with a limited set of drugs and that other *Candida* species or strains might behave differently with those drugs.

## 5. Conclusions

Our study emphasises that with fungistatic agents, cells can remain active even at MIC levels. Although the growth of these cells would not be sufficient to create a colony on an antifungal-containing medium, it is undoubtedly sufficient to promote a relapse at a later stage. In this context, the use of fungicidal agents (namely amphotericin) could be a valuable option, as in our experiments only amphotericin reduced the amount of *Candida* CFUs to close to 0. Indeed, several studies have recognized the efficacy of either intravenous administration [38] or bladder irrigation with amphotericin [34] for *Candida* UTIs despite potential counter-indications. Similar observations were made with vulvovaginal candidiasis (VVC), where amphotericin has been used in cases of recurrence [53] or treatment failure with azole compounds [54] and shows much lower resistance [55]. Indeed, our approach could be used for VVC, as artificial vaginal fluid mediums have been described and can be made readily [56].

The use of IMC does provide insight on such growth and activity and also allows the mechanisms allowing such growth (i.e., autophagy herein) to be hypothesized. Autophagy or the presence of dead cell debris and related material certainly deserves more attention, as the process is not restricted to dead biomass but also living biomass.

## Figures and Tables

**Table 1:** Growth rate (µ), doubling time (dt) and duration of the lag phase (λ) determined using conventional plate count and isothermal microcalorimetry data. Note the good correlation between µ CFU and µ IMC (R^2^ = 0.95, n = 15, *p* < 0.05).

	µ CFU	µ IMC	dt CFU	dt IMC	λ IMC	n
Control	0.147 ± 0.050	0.078 ± 0.003	4.7 ± 1.6	8.9 ± 0.3	4.1 ± 0.6	4
Amphotericin B	0.077 ± 0.005	0.037 ± 0.004	9.0 ± 0.6	18.9 ± 2.2	172.3 ± 4.3	4
Flucytosine	0.058 ± 0.001	0.022 ± 0.009	11.9 ± 0.2	31.5 ± 13.6	165.4 ± 2.9	3 *
Fluconazole	0.018 ± 0.001	0.014 ± 0.002	38.5 ± 2.1	49.5 ± 6.1	6.4 ± 6.2	4

* Thermal equilibrium was not properly achieved for one sample.

## Data Availability

The data presented in this study are available on request from the corresponding author.
